# Analysis of Postural Control in Patients Diagnosed with Unilateral Knee Osteoarthrosis and Its Relationship with the Risk of Falls

**DOI:** 10.1155/2023/5536304

**Published:** 2023-10-03

**Authors:** Robson Emiliano José de Freitas, Jaqueline Gleice Aparecida de Freitas, Carolina Pereira Vieira, Daniela Cristina Endres, Fábio Martins Inacio, Fernanda Grazielle da Silva Azevedo Nora

**Affiliations:** ^1^Orthopedics and Traumatology, IOG–Orthopedic Institute of Goiânia, Goiânia, Brazil; ^2^UEG–State University of Goiás, Goiânia, Brazil; ^3^Orthopedics and Traumatology, CRER–State Center for Rehabilitation and Readaptation Dr. Henrique Santillo, Goiânia, Brazil; ^4^UFG–Federal University of Goiás, Avenida Esperança s/n, Campus Samambaia, Goiânia, GO, Brazil

## Abstract

**Introduction:**

Knee osteoarthrosis, whether subtle or marked, appears to alter the stability and performance of the knee joint in activities of daily living that prevent the maintenance of bipedal posture. However, there is still a gap in the literature as to how knee osteoarthritis can affect static balance.

**Objective:**

To analyze the performance of postural control in elderly diagnosed with unilateral knee osteoarthrosis.

**Materials and Methods:**

40 elderly people of both sexes participated in this study, divided into two groups containing 20 elderly each. Group 1 (*G*1) consists of elderly patients who have received a diagnosis of unilateral knee osteoarthritis. Despite undergoing conservative treatment, their condition has shown insufficient improvement, leading to a clinical recommendation for total knee arthroplasty (TKA). The *G*2 group was made up of 20 elderly with an average age of 71.09 years, considered active, who do not have a diagnosis of osteoarthritis in the knee joint and practice physical activity. With the aid of a Baroscan pressure platform, the center of pressure (COP) displacement in the anteroposterior (COPAP) direction and mediolateral direction (COPML) and the area of center of pressure displacement were evaluated during bipedal postural control with eyes open and eyes closed.

**Results:**

During postural control with eyes open and eyes closed, the *G*1 group showed greater displacement of the COP in the anteroposterior direction—COPAP (*p* = 0.007)—and mediolateral direction—COPML (*p* = 0.033)—when compared to the *G*2 group. As for the area of displacement of the COP, group *G*1 presented a larger area of displacement (*p* = 0.002) than group *G*2 during bipedal postural control with open eyes. For the condition with eyes closed, both groups showed similar behaviors, which resulted in no present statistically significant differences.

**Conclusion:**

The results suggest that unilateral knee osteoarthritis influences bipedal postural control and activities of daily living that require this static balance, since information from the somatosensory system is reduced, resulting in stability of tasks that require body control and promoting the risk of falls. From a clinical perspective, the results suggest that the assessment of bipedal postural control can assist orthopedic physicians in assessing joint stability in patients with unilateral knee osteoarthrosis.

## 1. Introduction

Knee osteoarthritis, considered a consequence of degeneration of the articular cartilage and subchondral bone, is a common clinical condition among the elderly, with the knee being the most affected load bearing joint [1]. It manifests itself as pain, joint discomfort, functional disability, and weakness of the knee flexor and extensor muscles, in addition to alignment disorders resulting from the loss of articular cartilage and ligament changes [2]. Therefore, this is a chronic-degenerative, slowly progressive, and idiopathic disease of the knee joint, which affects the elderly [1, 2].

Therefore, aging is often associated with an increased risk of falls in the elderly. As people age, many changes occur in the body that can affect stability, balance, and coordination. Furthermore, factors such as osteoarthritis, use of certain medications, and changes in the environment can contribute to an increased risk of falls in the elderly [1, 2].

Osteoarthritis significantly heightens the risk of falls among elderly individuals, primarily attributable to its adverse impact on joint functionality, mobility, and balance. The degradation of cartilage within osteoarthritis-affected joints can engender alterations in a person's movement patterns and their capacity to support body weight, ultimately leading to an unstable and modified gait [1–3]. Consequently, the diminished range of motion within the knee joint can exert a substantial influence on gait mechanics and the ability to adapt to irregular surfaces or obstacles. These changes, in turn, disrupt the individual's equilibrium, impeding postural control and rendering everyday activities such as walking or rising from a chair more challenging [3, 4]. The presence of osteoarthritis can significantly compromise balance and increase the vulnerability to falls among the elderly population by influencing joint function, mobility, and gait patterns during routine activities.

Conservative treatment aims to effectively manage symptoms associated with knee osteoarthritis, including pain, reduced muscle strength, and limited mobility. This approach involves a combination of strategies, including lifestyle adjustments, medication, professional guidance, and the prescription of targeted physical exercises. During the initial phase of the disease, treatment is often elective, with a focus on noninvasive measures. These may include physiotherapy, the use of chondroprotectors (substances that support joint health), and the importance of weight management. These interventions aim to alleviate discomfort and improve the patient's functional capacity. However, as the condition progresses and the patient's functional abilities decline, a more advanced intervention may become necessary [3, 4]. Total knee arthroplasty (TKA) is a surgical procedure designed to address the advanced stages of knee osteoarthritis. Its primary objectives are to restore the knee's range of motion, minimize or eliminate pain, and enhance the overall stability of the knee joint by addressing muscular and ligamentous concerns [3–5].

Consequently, when conducting studies focusing on postural control activities, whether static or dynamic, employing a plantar pressure platform, the primary parameter of interest is the center of pressure (COP). This is recognized as the point of application for the resultant vertical forces acting upon the support surface of the feet [6, 7]. The variables under investigation commonly include the area of COP displacement and two-dimensional COP velocities.

The behavior of the COP effectively represents the collective response of the postural control system to gravitational forces. It reflects the neuromotor response to oscillations of the center of mass (CM) [6, 7]. Importantly, it is distinct from the center of mass (CM) and, in cases where both feet are in contact with the ground, the resultant COP position is situated between the two feet. Its location is contingent upon the relative distribution of load between each foot. Consequently, understanding COP behavior is invaluable for evaluating postural stability during both static and dynamic activities. This understanding aids in identifying potential imbalances within the postural control system and is an invaluable resource for assessing the balance of patients diagnosed with hip joint or knee joint injuries [2–4].

In elderly, knee osteoarthritis exerts a notable influence on stability and balance. This impact arises from factors such as pain, inflammation, and alterations in joint biomechanics. Evaluating postural control provides valuable insights into how the disease affects both the musculoskeletal and neuromuscular systems, thereby enriching our academic understanding of this condition [8, 9]. Assessing postural control in elderly individuals with knee osteoarthritis offers the potential to pinpoint specific risk factors associated with impaired balance. Such investigations hold the promise of revealing critical insights into clinical or demographic characteristics that may serve as predictors for an elevated risk of falls or other complications [8, 9]. In a clinical context, the evaluation of postural control plays a pivotal role in determining the most effective therapeutic strategies for this population. By understanding how knee osteoarthritis impacts postural stability, healthcare professionals can tailor interventions to enhance patient outcomes and overall quality of life.

As static balance becomes a more challenging motor task for elderly individuals diagnosed with knee osteoarthritis, it is likely that differences in performance will be observed. These differences arise due to the impaired provision of somatosensory information resulting from the pathology. Consequently, the primary question addressed in this study was to investigate whether the performance of bipedal postural control in elderly individuals diagnosed with unilateral knee osteoarthritis undergoes changes as the disease progresses. The specific objective was to assess two critical parameters: the amplitude of center of pressure (COP) displacement and the area of COP displacement. This assessment was conducted in the bipedal position, both with eyes open and eyes closed. The study population consisted of elderly individuals diagnosed with unilateral knee osteoarthritis, and their performance was compared with that of an active elderly control group.

This study aims to contribute valuable insights into the capacity of elderly individuals with osteoarthritis to manage the gravitational field and maintain static balance while in a standing position. In the realm of clinical applications, this knowledge may serve as a reference point in the future. It could assist healthcare professionals in identifying physiological patterns and aid in making informed decisions regarding surgical and nonsurgical treatment options for individuals dealing with bipedal postural control challenges due to unilateral knee osteoarthritis.

## 2. Materials and Methods

This is a cross-sectional study in which the behavior of the center of pressure (COP) was analyzed during bipedal postural control in the elderly.

### 2.1. Participants

40 elderly people of both sexes participated in this study, divided into 2 groups. The *G*1 group comprised 20 elderly individuals, with a mean age of 71.59 years, all diagnosed with unilateral knee osteoarthritis. Despite attempting conservative treatments, this group was recommended for primary total knee arthroplasty (TKA) due to treatment ineffectiveness. Group *G*2, on the other hand, was made up of 20 elderly people with an average age of 71.09 years, considered active, who do not have a diagnosis of osteoarthritis in the knee joint and practice physical activity (hydrogymnastics) at least 3 times a week for more than a year, as shown in Table 1.

### 2.2. Ethical Aspects

The present study was approved by the Research Ethics Committee of the Federal University of Goiás (CEP/UFG) registered under opinion number 24845019.2.0000.5083. Only the elderly who completed and signed the Free and Informed Consent Form (TCLE) participated in the study, authorizing participation in the study and being able to withdraw participation at any stage of the study.

### 2.3. Postural Control

To evaluate bipedal postural control, a Baroscan pressure platform was used, which has an active area of 50 × 50 cm with a quantity of 4,096 capacitive sensors and is produced by the company HS Technology, Brazil.

The elderly was instructed to perform bipedal support, in which they were instructed to maintain a static posture on the Baroscan platform, with their arms resting at their sides, with their eyes open and closed. Three attempts were performed, randomly distributed in each condition. Each trial lasted 60 seconds, with a 60-second break between each trial.

The pressure platform provided information about the behavior of the center of pressure (COP) in the anteroposterior (COPAP) and mediolateral (COPML) directions. The signals from the Baroscan platform were collected at a frequency of 100 Hz.

**Figure 1:**
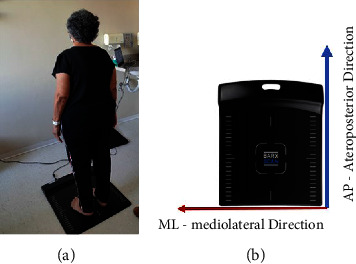
Postural control assessment of the present study. (a) Positioning of the elderly during postural control with eyes open and closed. (b) Baroscan platform indicating the direction of COP displacement: anteroposterior (AP) and mediolateral (ML).

The elderly was advised if they felt any discomfort that prevented them from continuing the test, such as dizziness, anxiety, or something that bothered them, simply letting them know that the activity had been interrupted. For group *G*1, the assessment of bipedal postural control was carried out 1 hour before the surgical procedure, and for group *G*2, it was carried out 1 hour before their hydrogymnastics class.  Figure 1 shows the assessment of postural control proposed in the present study.

### 2.4. Variables Analyzed

The center of pressure (COP) is represented by a two-dimensional position vector, which serves as an immediate indicator of the point where the ground reaction force is generated. This force results from the contact between the body and the supporting surface, offering a dynamic representation of how this force displacement changes over time [6, 7].

To analyze the COP effectively, it was essential to break it down into two distinct components or directions, known as the mediolateral (ML) and anteroposterior (AP) directions. As a result, the calculation of COP oscillation amplitude in both these directions, expressed in centimeters (cm), involved measuring the distance between the maximum and minimum positions observed in the mediolateral and anteroposterior directions [6, 7].

### 2.5. Statistical Analysis

Statistical analysis was performed using Minitab Statistical Software Version 21.0. After verifying the normality of the distributions and the homogeneity of the data using the Kolmogorov–Smirnov test, the nonparametric test, Kruskal–Wallis test, was applied to verify differences between the group diagnosed with knee arthrosis and submitted to primary TKA (*G*1) and the hydrogymnastics group (*G*2) for the selected variables. A significance level of *p* ≤ 0.05 was used as a statistical reference. Variables will be presented as mean values.

## 3. Results

The general objective of this study was to investigate postural control in the bipedal position of elderly people reported with unilateral knee osteoarthritis and elderly people who practice water aerobics. Based on the results, the effect of knee osteoarthrosis during postural control was evident, providing greater proprioceptive information for maintaining static balance when compared to another group of elderly people.

Therefore, Table 2 presents the results of the COP displacement amplitude in the anteroposterior (COPAP) and mediolateral (ML) directions during bipedal postural control, under two conditions: eyes open (AO) and eyes closed (OF).

For the bipedal postural control with eyes open and eyes closed, the *G*1 group showed significantly higher values (*p*=0.007) for the COP displacement amplitude in the anteroposterior direction (COPAP) when compared to the *G*2 group. In the range of displacement of the COP in the mediolateral direction (COPML), the *G*1 group presented significantly higher values when compared to the *G*2 group (*p*=0.033).

Table 3 shows the COP displacement area during bipodal postural control in the open-eye condition and in the closed-eye condition.

For the area of displacement of the COP (area) during the open-eye bipedal postural control, the *G*1 group presents statistically higher values than the *G*2 group (*P*=0.002). As for the displacement area of the COP during the closed-eye bipedal postural control, there were no statistically significant differences.

## 4. Discussion

Aging presents challenges in maintaining physical health, muscle strength, and balance. Pathological conditions such as osteoarthrosis can affect quality of life and increase physiological and psychological changes. Therefore, the present study compared the postural stability of two groups of elderly people: elderly having unilateral knee osteoarthrosis (*G*1) and healthy elderly practicing water aerobics (*G*2) during postural control, with eyes open and eyes closed.

During postural control with eyes open and eyes closed, the group of elderly people diagnosed with unilateral knee osteoarthrosis (*G*1) showed a greater range of displacement of the center of pressure in the anteroposterior direction (COPAP) when compared to the group of healthy elderly people practicing hydrogymnastics (*G*2). Elderly people diagnosed with unilateral knee osteoarthritis present delayed anticipatory postural adjustments, leading to slower voluntary movements and an increased risk of falls. Therefore, active elderly people have less displacement of the COPAP, as regular physical activity contributes to a better ability to maintain stability posture in challenging everyday situations. Therefore, changes in the muscular strength of the knee joint alter the range of COPAP displacement, consequently generating joint instability and increasing the risk of falls [10–12].

When analyzing the displacement of the center of pressure in the mediolateral direction (COPML) during postural control with eyes open and eyes closed, the *G*1 group showed greater displacement when compared to the *G*2 group. Therefore, elderly people diagnosed with unilateral knee osteoarthritis (*G*1) have functional limits of stability, which leads to more precarious stability conditions when compared to healthy active elderly people (*G*2). Therefore, this change suggests that the *G*1 group has difficulty regaining balance during activities of daily living, thus causing greater postural instability [12–14].

Therefore, hydrogymnastics is a physical activity that contributes to improving balance, muscle strength, flexibility, and motor coordination, thus reducing the risk of falls. The resistance of the water offers natural resistance during exercises, making them more effective in strengthening the muscles of the lower limb, which play a fundamental role in maintaining balance and preventing falls [15–21]. It also allows greater range of movement due to reduced resistance in the water, which can improve flexibility and reduce muscle and joint stiffness [15–21]. Finally, the aquatic environment provides a feeling of safety, as elderly people have fewer worries about serious injuries resulting from falls. This can help reduce the fear of falling and thus encourage regular participation.

As for the COP displacement area (area), the group of elderly people diagnosed with unilateral knee osteoarthritis (*G*1) only presented statistically higher values than the group of active healthy elderly people (*G*2) only during postural control with open eyes. Therefore, the *G*2 group has a greater ability to maintain stability in a smaller area during static balance tasks. Therefore, regular physical activity helps to improve proprioception, muscular strength, motor coordination, and postural stability, allowing healthy elderly people to maintain postural stability more safely during activities of daily living, thus promoting a lower risk of falls [22–24].

Therefore, the base of support represented by the variable area in elderly people diagnosed with unilateral knee osteoarthritis tends to be broader, while in healthy elderly people, it may be narrower, reflecting differences in levels of physical activity and the development of postural stability skills. Because the base of support is a dynamic variable that adjusts to the specific demands of static and sound balance tasks during activities of daily living [6, 7]. Adequate postural stability is essential for preventing falls and maintaining function in the elderly, regardless of their level of physical activity [1–3, 8, 9, 22–24].

Thus, successful aging and maintaining postural stability are complex challenges for elderly people diagnosed with unilateral knee osteoarthritis. Understanding these challenges is important to recognize that treatment aimed at elderly people diagnosed with osteoarthritis must aim to preserve joint function, promote improvement in muscle strength and joint stability, and improve static balance.

## 5. Conclusion

The results of this study suggest an effect of knee osteoarthritis on postural control when compared with healthy and active elderly people. This fact was noticed in tasks that deactivate bipedal support with eyes open and eyes closed, suggesting that deficiencies in postural control can be observed. A possible explanation for this deficiency may be related to reduced proprioceptive information.

In the clinical context, the assessment of postural control serves as a valuable complementary diagnostic tool for orthopedic doctors. It aids in the comprehensive evaluation of knee joint stability and the assessment of fall risk, enabling more informed decisions regarding surgical intervention, especially when elderly patients do not respond positively to nonsurgical treatment options.

## Figures and Tables

**Table 1:** Characteristics of participants.

Variables	*G*1	*G*2
Age (years)	71.59 (±14.09)	71.09 (±13.08)
Gender (male/female)	5/11	4/16
Weight (kg)	80.63 (±9.71)	50.44 (±6.11)
Height (cm)	162.91 (±0.09)	159.38 (±1.81)
EVA^*∗*^	10	—

*G*1 = elderly people diagnosed with unilateral knee osteoarthritis; *G*2 = active elderly people without a diagnosis of unilateral osteoarthrosis in the knee. ^*∗*^The EVA scale was applied only to the group that was diagnosed with arthrosis. Data are presented as mean values and ± standard error.

**Table 2:** COP displacement amplitude during postural control with eyes open and eyes closed.

Conditions	Variables	*G*1	*G*2	*P* value
Eyes open	COPAP (cm)	2.41 (±1.34)	1.36 (±0.54)	**0.007** ^*∗*^
	COPML (cm)	2.07 (±1.80)	1.02 (±0.43)	**0.033** ^*∗*^

Eyes closed	COPAP (cm)	6.25 (±0.68)	3.48 (±0.76)	**0.002** ^*∗*^
	COPML (cm)	4.81 (±0.52)	3.93 (±1.10)	0.430

*G*1 = elderly people diagnosed with unilateral knee osteoarthritis; *G*2 = active elderly people without a diagnosis of unilateral osteoarthrosis in the knee. ^*∗*^Significant Kruskal–Wallis test (*p* < 0.05). Data are presented as mean values and ±standard error. In the table, values that present statistical significance are in bold.

**Table 3:** COP displacement area during bipedal postural control with eyes open and eyes closed.

Conditions	Variables	*G*1	*G*2	*P* value
Eyes open	Area (cm^2^)	1.18 (±0.94)	1.18 (±0.94)	**0.002** ^*∗*^
Eyes closed	Area (cm^2^)	3.92 (±1.10)	3.92 (±1.10)	0.430^*∗*^

*G*1 = elderly people diagnosed with unilateral knee osteoarthritis; *G*2 = active elderly people without a diagnosis of unilateral osteoarthrosis in the knee. ^*∗*^Significant Kruskal–Wallis test (*p* < 0.05). Data are presented as mean values and ±standard error. In the table, values that present statistical significance are in bold.

## Data Availability

The data used to support the findings of this study are included within the article.
